# Left ventricular shape predicts arrhythmic risk in fibrotic dilated cardiomyopathy

**DOI:** 10.1093/europace/euab306

**Published:** 2021-12-15

**Authors:** Gabriel Balaban, Brian P Halliday, Daniel Hammersley, Christopher A Rinaldi, Sanjay K Prasad, Martin J Bishop, Pablo Lamata

**Affiliations:** Department of Biomedical Engineering, School of Biomedical & Imaging Sciences, King’s College London, 249 Westminster Bridge Road, SE1 7EH London, UK; Biomedical Informatics Group, Department of Informatics, University of Oslo, Oslo, Norway; Department of Computational Physiology, Simula Research Laboratory, Oslo, Norway; PharmaTox Strategic Research Initiative, Deparment of Pharmacy, University of Oslo, 0373 Oslo, Norway; Cardiovascular Magnetic Resonance Unit, Royal Brompton Hospital, London, UK; National Heart and Lung Institute, Faculty of Medicine, Imperial College London, London, UK; Cardiovascular Magnetic Resonance Unit, Royal Brompton Hospital, London, UK; National Heart and Lung Institute, Faculty of Medicine, Imperial College London, London, UK; Department of Biomedical Engineering, School of Biomedical & Imaging Sciences, King’s College London, 249 Westminster Bridge Road, SE1 7EH London, UK; Department of Cardiology, St Thomas’ Hospital, London, UK; Cardiovascular Magnetic Resonance Unit, Royal Brompton Hospital, London, UK; National Heart and Lung Institute, Faculty of Medicine, Imperial College London, London, UK; Department of Biomedical Engineering, School of Biomedical & Imaging Sciences, King’s College London, 249 Westminster Bridge Road, SE1 7EH London, UK; Department of Biomedical Engineering, School of Biomedical & Imaging Sciences, King’s College London, 249 Westminster Bridge Road, SE1 7EH London, UK

**Keywords:** Non-ischaemic dilated cardiomyopathy, Cardiac magnetic resonance imaging, Arrhythmias, Machine learning, Shape analysis, Risk stratification

## Abstract

**Aims:**

Remodelling of the left ventricular (LV) shape is one of the hallmarks of non-ischaemic dilated cardiomyopathy (DCM) and may contribute to ventricular arrhythmias and sudden cardiac death. We sought to investigate a novel three dimensional (3D) shape analysis approach to quantify LV remodelling for arrhythmia prediction in DCM.

**Methods and results:**

We created 3D LV shape models from end-diastolic cardiac magnetic resonance images of 156 patients with DCM and late gadolinium enhancement (LGE). Using the shape models, principle component analysis, and Cox-Lasso regression, we derived a prognostic LV arrhythmic shape (LVAS) score which identified patients who reached a composite arrhythmic endpoint of sudden cardiac death, aborted sudden cardiac death, and sustained ventricular tachycardia. We also extracted geometrical metrics to look for potential prognostic markers. During a follow-up period of up to 16 years (median 7.7, interquartile range: 3.9), 25 patients met the arrhythmic endpoint. The optimally prognostic LV shape for predicting the time-to arrhythmic event was a paraboloidal longitudinal profile, with a relatively wide base. The corresponding LVAS was associated with arrhythmic events in univariate Cox regression (hazard ratio = 2.0 per quartile; 95% confidence interval: 1.3–2.9), in univariate Cox regression with propensity score adjustment, and in three multivariate models; with LV ejection fraction, New York Heart Association Class III/IV (Model 1), implantable cardioverter-defibrillator receipt (Model 2), and cardiac resynchronization therapy (Model 3).

**Conclusion:**

Biomarkers of LV shape remodelling in DCM can help to identify the patients at greatest risk of lethal ventricular arrhythmias.

## Graphical Abstract

**Graphical Abstract:**
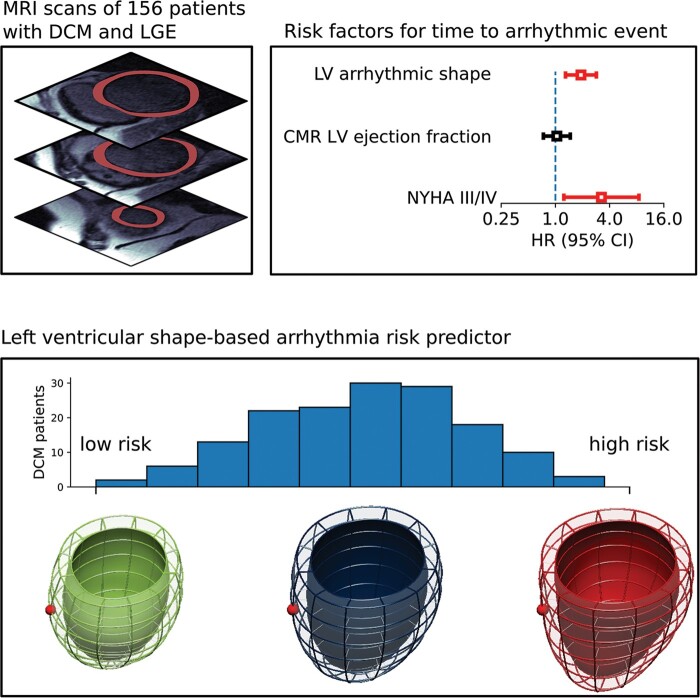

**What’s new?**

- We applied a novel computational anatomy approach to retrospectively assessing the left ventricular (LV) shape of 156 dilated cardiomyopathy patients with late gadolinium enhancement on cardiac magnetic resonance.
- Using a Cox–Lasso technique, we derived a novel shape score (LV arrhythmic shape) constructed from the geometrical modes of variation, which was independently predictive of arrhythmic events.

## Introduction

Non-ischaemic dilated cardiomyopathy (DCM) is a heterogenous condition, characterized by several morphological and pathological changes, including ventricular cavity dilation, myocardial wall thinning, and fibrosis.^1^ Unfortunately, DCM is also associated with a high risk of ventricular arrhythmias. Implantable cardioverter-defibrillators (ICDs) can reduce the risk of sudden cardiac death (SCD) in patients with DCM.^2^ However, these devices are expensive and associated with complications. Only a small proportion of patients with a device receive an appropriate shock^2^ under the current ejection fraction and New York Heart Association (NYHA) class criteria.^1^ Techniques that can identify those at highest risk of SCD and who are most likely to gain longevity are required to improve the appropriate deployment of ICD therapy.

Recent work has shown that the presence of mid-wall fibrosis, evidenced by late gadolinium enhancement (LGE) on cardiac magnetic resonance (CMR), predicts major ventricular arrhythmias and SCD.^3,^^4^ While the absence of LGE confers a low risk of SCD events, the positive predictive value of LGE remains sub-optimal when considering the cost and lifelong impact of ICD implantation. Not all DCM patients with positive LGE findings will experience an arrhythmic,^3,^^4^ event, and thus better methods to identify the patients at highest risk of arrhythmia are therefore required.

The most readily identifiable anatomical change in DCM is the morphological dilation of the left ventricular (LV) cavity that forms the basis for the diagnosis. Previous analysis suggests that myocardial wall thinning,^5^ thick-to-thin gradients,^5^ and overall tissue surface area^6^ may form part of an arrhythmic substrate, enhancing risk. However, such complex remodelling patterns are difficult to characterize with simple clinical image-based measurements. An automated way of characterising and measuring these subtle myocardial remodelling patterns may therefore lead to improved risk stratification.

In this study, we hypothesize that LV shape changes due to remodelling contain prognostic markers related to the risk of future arrhythmias in DCM patients with LGE. While prior studies have focused on global LV shape metrics, such as sphericity and cavity volume^7,^^8^; here, we search for a shape-based prognostic marker with machine learning techniques applied to 3D LV computational anatomy models derived from CMR.

## Methods

### Study population

This is a retrospective study of 156 patients who were enrolled in the Royal Brompton Hospital Cardiovascular Biobank Project between 2002 and 2015 during CMR evaluation. All patients gave written informed consent, and the study was approved by the UK National Research Ethical Committee and performed in accordance with the Declaration of Helsinki. All patients referred to the Royal Brompton Hospital for CMR for the evaluation of NIDCM between 2002 and 2015 were considered. Patients were included if they had dilated cardiomyopathy, as identified by increased LV end-diastolic volume indexed to body surface area and reduced LVEF compared with published reference ranges normalized for age and sex, as well as evidence of mid-wall or subepicardial LGE on CMR in two orthogonal planes and two phase encoding directions, as well as having signed a consent form allowing for data use across institutions. Patients were excluded if they had ischaemic heart disease defined as stenosis >50% in a major coronary artery, inducible ischaemia on functional testing or subendocardial or transmural LGE indicative of previous myocardial infarction. Further exclusion criteria were hypertensive heart disease, primary valvular disease, athletic remodelling, congenital heart disease, iron overload, and cardiac sarcoidosis. A single patient experienced an arrhythmic episode prior to baseline; no other patients had prior recorded ventricular tachycardia (VT) or ventricular fibrillation (VF).

### Follow-up, endpoint, and device implantations

Patients were followed-up from baseline using questionnaires and telephone interviews, and by gathering information from family physicians, cardiologists, and hospital records. Follow-up time was calculated from the baseline CMR scan until an endpoint occurred or last contact with the patient. All events were adjudicated by an independent committee blinded to the CMR results.

The study endpoint was a composite of SCD, aborted sudden cardiac death (ASCD), and sustained VT. Sudden cardiac death was defined as unexpected death either within 1 h of cardiac symptoms in absence of progressive cardiac deterioration, during sleep, or within 24 h of last being seen alive and well. Implantable cardioverter-defibrillator and cardiac resynchronization therapy (CRT) devices were implanted during follow-up on the basis of guideline recommendations. Those implanted outside guidelines were discussed in a multidisciplinary team meeting. All-cause mortality and heart failure outcomes (pump failure) were not considered in the study endpoint.

### Cardiac magnetic resonance acquisition and segmentation

**Figure 1:**
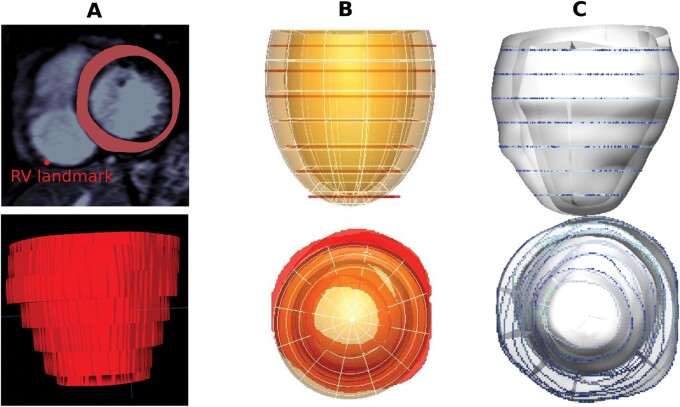
Methodology to capture the LV shape. (*A*) Top: CMR image with segmented myocardium and RV landmark to break the LV symmetry. Bottom: 3D volumetric representation of short-axis contours. (*B*) An idealized ellipsoid (transparent orange) tailored to fit the length and diameter of the contours (red). (*C*) Result of fitting process with points of the contours colour coded according to the distance to the nearest epicardial contour (rainbow scale, from blue—0 cm to red—1 cm). 3D, three-dimensional; CMR, cardiac magnetic resonance; LV, left ventricular.

Short-axis CMR images were acquired with a scanning resolution of 1.3–2.2 mm in plane and with a 7–10.5 mm slice thickness using a previously described protocol.^3^ The contours of the epi and endocardium were semi-automatically segmented (CVI42, Circle Cardiovascular Imaging Inc.) by an expert blinded to the patient outcomes. Areas of LGE were identified using a semi-automated full-width at half maximum approach (CVI42) by two independent observers blinded to patient outcomes. An additional point was marked, where the most basal part of the RV epicardium meets the liver (*Figure 1*). This point was required to break the circumferential symmetry of the LV in the shape models and provided a marker for the most basal slice. Images above the most basal RV slice, including at the LV valve plane, were removed.

### Three-dimensional left ventricular shape reconstruction from cardiac magnetic resonance

Each patient’s LV end-diastolic shape was reconstructed from short-axis image contours by building smooth, tailored LV 3D meshes.^9^ In brief, an idealised ellipsoid (with 96 cubic Hermite elements, 194 nodes and 4656 degrees of freedom) was fitted to each patient’s epicardial and endocardial contours (*Figure 1*), using image registration and mesh warping techniques.^9^ All 156 reconstructed LV geometries were then spatially aligned by their centres of mass, and oriented by two directions: apex to base (perpendicular to MRI slice plane) and left to right (LV centre of mass to RV landmark). All LV geometries are available via the FigShare repository: https://doi.org/10.6084/m9.figshare.11417295. Custom Matlab code for shape reconstruction is available on request from https://github.com/PabloLamata/ComputationalAnatomy.

### Left ventricular geometry measurements

Geometrical measurements were used to describe each patient’s reconstructed LV shape. These measurements were extracted from the 3D meshes and consisted of end-diastolic volume (BPvol), length (L), mean wall thickness, and sphericity. Sphericity was calculated by comparing BPvol to the volume of a sphere with diameter equal to the LV length (L).

$$\text{Sphericity}=\text{BPvol }\left(\text{4}/\text{3}\right)\;\pi \;{\left(\text{L}/\text{2}\right)}^{\text{3}}.$$

### Machine learning and statistical analysis

Each patient’s LV shape was described by a vector *X_i_* of length 4656, which parametrized the geometry of each patient’s fitted LV ellipsoid. All patient LV parameters were then combined in a matrix, *X* = *[X_1_ …. X_N_*_=_*_156_]*, and analysed with principal component analysis (PCA). Principal component analysis generated a set of modes M_j_ of LV shape variation, where each M_j_ was a vector in the 4656 dimensional shape space, and the M_j_ were ordered from most to least explained shape variance. Each patient’s LV geometry could then be related to the average geometry $\bar{X}$, and a reduced set of PCA modes, modes 1–10 (chosen because they capture the most shape variance), and their coefficients *c_ij_*:

$$X_{i}\approx \bar{X}+\sum_{j=1}^{10}c_{ij}M_{j}.$$

Here, the coefficients *c_ij_* quantify the relative contribution of each shape mode to each patients’ LV geometry, and can be interpreted as potential biomarkers. An optimal linear combination of modes was learned via penalized Cox–Lasso regression with the arrhythmic endpoint (Supplementary material online, *Methods* for details), using a cross-validated likelihood score. The LVAS score was computed for each patient as a linear combination of their PCA coefficients using the weights learned in the Cox–Lasso regression. The LVAS scores were max–min normalized to the range (0–1), with a higher LVAS indicating a worse shape with a greater risk of arrhythmia. Access to the functionality to compute the LVAS score is provided through a web-service available in the Cardiac Atlas Project from ‘http://www.cardiacatlas.org/’.

Cox proportional hazards regression was used to evaluate LVAS and other variables as risk predictors. A propensity score adjustment of LVAS was performed to control for potential confounding by baseline clinical variables. The LVAS propensity score was calculated by a non-parametric method (see Supplementary material online for details) with input variables selected by the criteria: no missing data and Cox univariate *P*-value < 0.25, similar to.^10^ The LVAS propensity score and LVAS were used in two Cox regression models to obtain adjusted hazard ratios for the LVAS-arrhythmia association, using inverse probability weighing and covariate adjustment.

Smoking status, history of atrial fibrillation, and mitral regurgitation data contained missing entries. Multiple imputation by chained equations was used to estimate the association of these variables with the arrhythmic endpoint (see Supplementary material online for details).

Baseline variables were compared using Mann–Whitney tests for continuous variables and *χ*^2^ tests for categorical variables, using all available data for variables with missing entries. Correlations between variables were assessed using Spearman *ρ* coefficients along with two-sided *t*-tests for significance. A *P*-value of <0.05 was considered significant. Python 3.6.9 and the packages statsmodels, scipy, autoimpute, and lifelines were used for statistical analysis.

## Results

### Baseline characteristics, arrhythmic events, and device implantations

Baseline characteristics of the study cohort are shown in *Table 1*. A total of 156 patients were included, with a median and interquartile range (IQR) age 58.0 (IQR: 19.3) years, 82.1% male, and median LVEF 38% (IQR: 18.2%). The patients were followed up for a median of 7.7 (IQR: 3.9) years, during which 25 patients experienced an arrhythmic event. Specifically, 15 patients had sustained VT, 8 patients had ASCD, and 2 patients suffered SCD. The patients who experienced an event had a worse LVAS score (median LVAS = 0.71 vs. 0.57, *P* < 0.01). No significant differences between event and no event groups were found with regards to other baseline variables (*Table 1*) or DCM aetiology (Supplementary material online, *Table S1*). Over the course of follow-up, 81 (51.9%) patients received an ICD, 45 (28.8%) of whom also received cardiac resynchronization therapy. The relative timings of ICD implantations and events are shown in Supplementary material online, *Figure S1*. All ICD implantations were for primary prevention with the exception of a single patient who received their device for secondary prevention following an arrhythmic episode prior to baseline.

**Table 1:** Baseline patient characteristics

Baseline variables	All (*N* = 156)	No event (*N* = 131)	Event (*N* = 25)	*P*
LVAS (0–1)	0.53 (0.31)	0.51 (0.33)	0.65 (0.23)	>0.001 ^*^
Age (years)	58.0 (19.3)	58.9 (19.3)	52.8 (14.5)	0.18
Male sex	128 (82.1%)	107 (81.7%)	21 (84.0%)	0.99
Body surface area (m^2^)	2.0 (0.3)	2.0 (0.3)	2.0 (0.3)	0.88
Heart rate (b.p.m.)	71.0 (21.3)	71.0 (22.0)	70.0 (18.0)	0.28
Systolic blood pressure (mmHg)	123.0 (27.2)	124.0 (27.5)	120.0 (20.0)	0.63
Diastolic blood pressure (mmHg)	72.5 (18.2)	73.0 (17.5)	72.0 (19.0)	0.85
Moderate alcohol excess	25 (16.0%)	18 (13.7%)	7 (28.0%)	0.14
Non-smoker^a^	77 (49.4%)	67 (51.1%)	10 (40.0%)	0.40
Smoker^a^	15 (10.1%)	11 (8.8%)	4 (17.4%)	–
Ex-smoker^a^	56 (37.8%)	47 (37.6%)	9 (39.1%)	–
Comorbidity				
History of atrial fibrillation^a^	43 (32.3%)	38 (33.9%)	5 (23.8%)	0.45
Mitral regurgitation^a^	88 (57.5%)	75 (58.6%)	13 (52.0%)	0.66
Diabetes	22 (14.1%)	18 (13.7%)	4 (16.0%)	0.99
Left bundle branch block	37 (23.7%)	31 (23.7%)	6 (24.0%)	0.83
NYHA functional class				
I/II	129 (82.7%)	111 (84.7%)	18 (72.0%)	0.21
III/IV	27 (17.3%)	20 (15.3%)	7 (28.0%)	–
Medications				
Beta-blocker	128 (82.1%)	105 (80.2%)	23 (92.0%)	0.26
Ace inhibitor/angiotensin receptor blocker	137 (87.8%)	114 (87.0%)	23 (92.0%)	0.72
Aldosterone antagonist	72 (46.2%)	63 (48.1%)	9 (36.0%)	0.37
Loop diuretic	86 (55.1%)	73 (55.7%)	13 (52.0%)	0.90
CMR measurements				
LV ejection fraction (%)	38.0 (18.2)	37.0 (18.0)	38.0 (20.0)	0.54
LV end-diastolic volume (mL)	255.2 (103.6)	256.6 (103.5)	242.8 (97.7)	0.9
LV end-diastolic volume index (mL/m^2^)	125.0 (53.2)	125.0 (54.5)	126.0 (50.0)	0.87
LV mass index (g/m^2^)	96.0 (37.2)	94.0 (38.0)	101.0 (31.0)	0.11
RV ejection fraction (%)	54.5 (18.2)	53.0 (19.5)	57.0 (10.0)	0.42
LGE volume (cm^3^)	7.0 (6.6)	6.5 (6.8)	8.5 (5.1)	0.26
LV geometry metrics				
Sphericity	0.63 (0.24)	0.63 (0.24)	0.67 (0.30)	0.38
Length (mm)	88.5 (17.1)	88.3 (17.4)	89.4 (15.2)	0.63
Mean wall thickness (mm)	7.4 (1.7)	7.3 (1.8)	7.6 (1.4)	0.51

Numerical data are median (IQR), compared with Mann–Whitney tests. Categorical data are frequency (%), compared with *χ*^2^ contingency tests.

LVAS, left ventricular arrhythmic shape score.

a Presence of missing data: smoking status 8 (5.1%), history of atrial fibrillation 23 (14.7%), and mitral regurgitation 3 (2%), compared with available data.

* *P* < 0.05.

### Patient three-dimensional left ventricular models and the average left ventricular anatomy in fibrotic dilated cardiomyopathy

**Figure 2:**
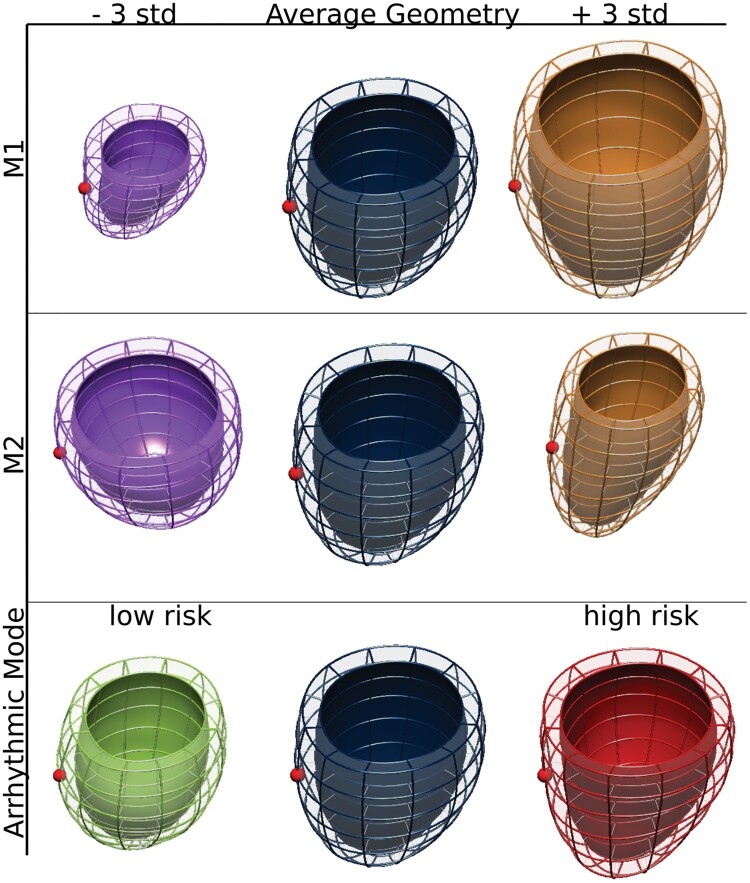
Modes of LV shape variation. The average patient LV geometry is shown in the middle column, and is shifted by 3 std along Modes 1 and 2, and the arrhythmic mode. The red dot points towards the manually selected RV landmark. LV, left ventricular.

Left ventricular ellipsoids were fitted to all 156 patient CMR scans with sub-voxel accuracy, with an average fitting error of 0.90 mm ± 0.42 mm, as measured by the distance from the fitted ellipsoid to the nearest CMR epicardial or endocardial contour. We observed a consistent linear relationship between the 3D model and CMR end-diastolic volumes (*R*^2^ = 0.83, Supplementary material online, *Figure*S2). An average LV geometry was created (*Figure 2*), which had an apex-to-base length of 85.46 mm, a cavity volume of 231.3 mL, an average wall thickness of 7.9 mm, and a sphericity of 1.63.

### Modes of left ventricular shape variation

**Figure 3:**
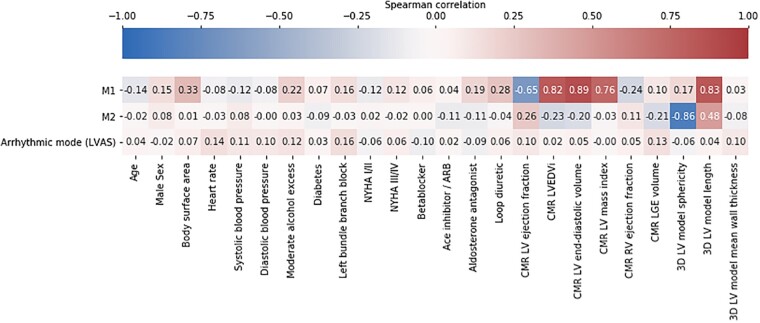
Spearman correlations between Modes 1 and 2 the arrhythmic mode and baseline variables. Correlations with absolute values ≥0.17 are statistically significant in a two-tailed *t*-test *P* < 0.05. M1 is significantly correlated with body surface area, moderate alcohol excess, aldosterone antagonist use, loop diuretic use, CMR LV ejection fraction, CMR LV ejection fraction index, CMR LV mass index, CMR RV ejection fraction, 3D LV model sphericity, and 3D LV model length. M2 is significantly correlated with CMR LV ejection fraction, CMR LV ejection fraction index, CMR LGE volume, 3D model sphericity, and 3D model LV length. The arrhythmic mode (LVAS) is not significantly correlated with any of the baseline variables. 3D, three-dimension; CMR, cardiac magnetic resonance; LGE, late gadolinium enhancement; LV, left ventricular; LVAS, left ventricular arrhythmic shape.

The fitted LV ellipsoids were decomposed into modes of variation using PCA. The first 10 PCA modes (named M1–M10) accounted for 88.30% of the anatomical variation observed in the cohort and were used in later analysis. *Figure 2* displays M1–2, whereas M3–10 are displayed in Supplementary material online, *Figure S3*. M1 was positively correlated with the CMR LV end-diastolic volume (*ρ* = 0.89, *P* < 0.001), 3D LV model length (*ρ* = 0.83, *P* = 0.001), and was negatively correlated with the CMR LV ejection fraction (*ρ* = −0.65, *P* < 0.001). M2 was most strongly correlated with 3D model LV sphericity (*ρ* = −0.86, *P* < 0.001), and 3D-model LV length (*ρ* = 0.48, *P* < 0.001). Further significant correlations between M1, M2, and other variables are shown in *Figure 3*.

### The arrhythmic left ventricular shape mode

**Figure 4:**
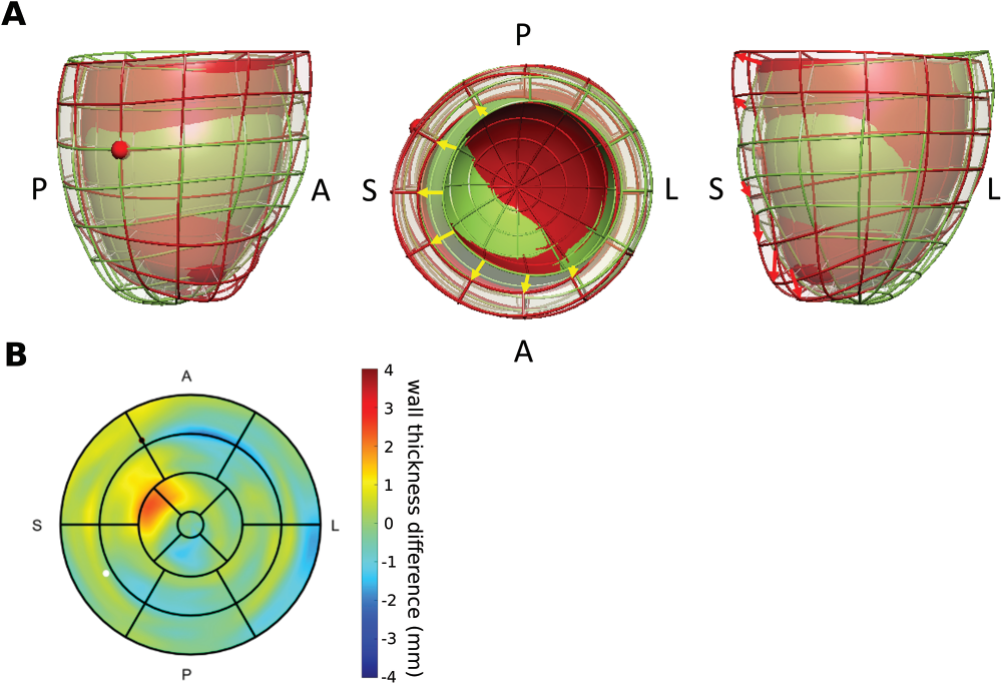
Detailed anatomical changes associated with the arrhythmic mode of variation. (*A*) Geometries representing plus (red) or minus (green) 3 std variation along the arrhythmic shape mode. The red sphere marks the direction towards the RV landmark, and the arrows highlight the deformation from the low- to high-risk LV morphology at the base (yellow arrows) and at the septum (red arrows). (*B*) Bullseye plot representation of the change in wall thickness when moving from the low- to the high-risk LV morphology, illustrating an irregular pattern with wall thinning in the basal lateral and the mid antero-lateral regions, and wall thickening in the septal apex. LV, left ventricular; RV, right ventricular.

Shape modes 5, 6, and 10, with corresponding coefficients (−0.018, 0.023, 0.037), formed the optimal combination for prognosticating the time to arrhythmic event, achieving the maximal cross-validated log-likelihood (Supplementary material online, *Figure S4*) of all the models selected by the Cox–Lasso method. The resulting arrhythmic mode is visualized in *Figure 2* (bottom row), and shows a characteristic ellipsoidal vertical cross-section (i.e. wider mid cavity compared to base) at the low-risk end of the patient distribution (−3 std), as compared to the more parabolic cross-section (i.e. wider basal to mid cavity diameter) at the high-risk end of the patient distribution (+3 std). Further anatomical details captured by the arrhythmic mode are shown in *Figure 4* and Supplementary material online, *Movie*. Interestingly, the arrhythmic mode had no significant correlations with any baseline variables (*Figure 3*), nor with six metrics related to each patient’s LGE pattern (Supplementary material online, *Figure S5*), originating from our previous study.^11^ The raw LVAS scores were within the range (−2.10 to 1.87), and were max–min normalized for downstream analysis.

**Figure 5:**
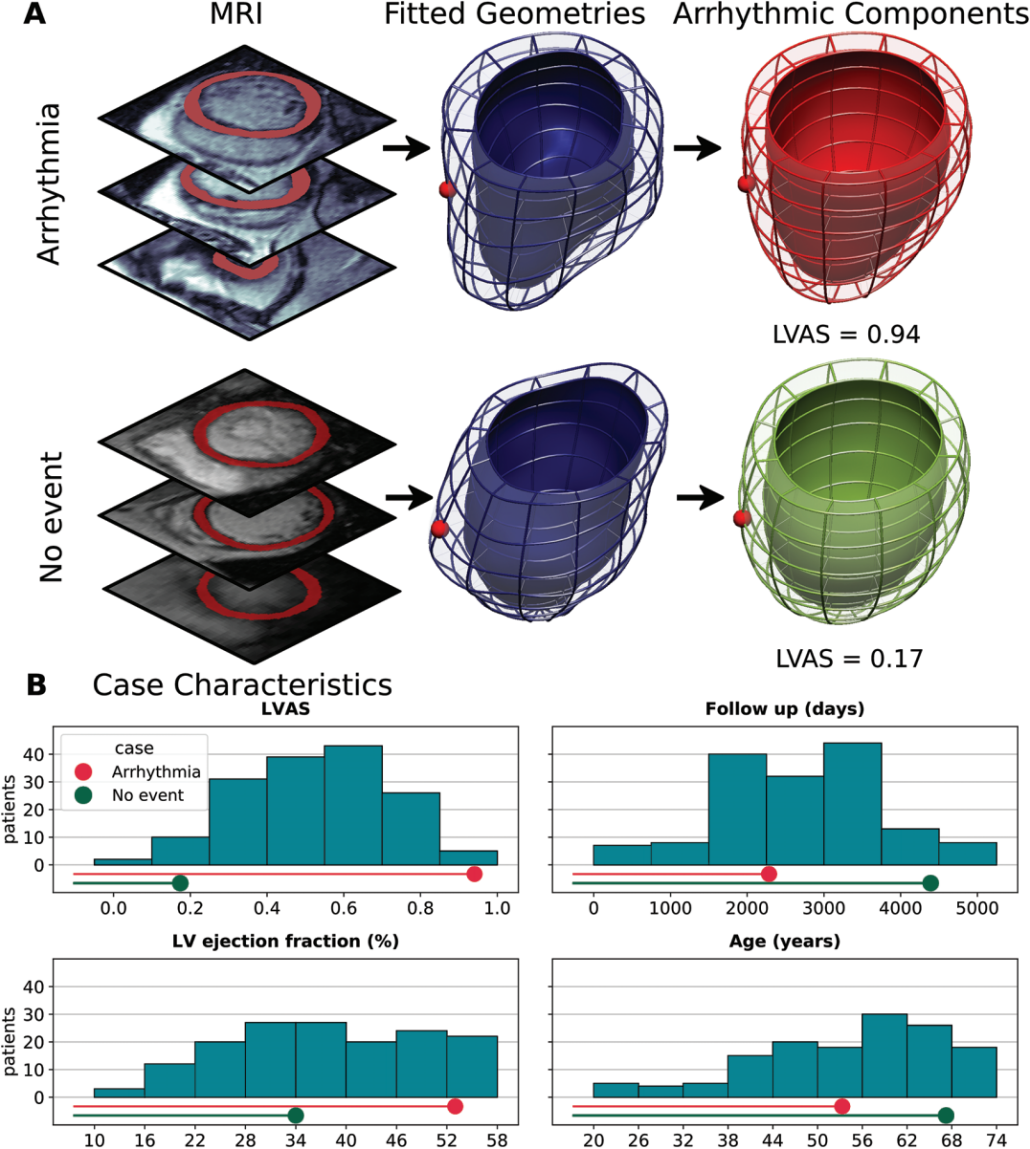
Arrhythmic shape analysis applied to two example patients. (*A*) LV arrhythmic shape analysis from two example patients, one of whom suffered an arrhythmic event during follow up. MRI images are a selection of each patients short-axis image stack. (*B*) Key characteristics of the two patients in comparison with each other and the entire cohort. The patient who met the arrhythmic end-point had a higher LVAS score, a greater LV ejection fraction, a lower NYHA class (1 vs. 3), and was younger than the patient who was event-free. LV, left ventricular; LVAS, left ventricular arrhythmic shape; MRI, magnetic resonance imaging; NYHA, New York Heart Association.

The potential utility of the arrhythmic mode is highlighted in *Figure 5*, which contrasts two patients with differing outcomes. One patient met the arrhythmic endpoint after 6.3 years, whereas the second was event-free for 12 years. The patient who suffered the arrhythmia had a better LV ejection fraction (54% vs. 34%), was younger (53.3 vs. 67.2 years), and was in a lower NYHA class (1 vs. 3), than the event-free patient. Nevertheless, the LVAS was elevated in the patient who suffered the arrhythmia (LVAS = 0.94 vs. 0.17).

### Time to arrhythmic event analysis

**Figure 6:**
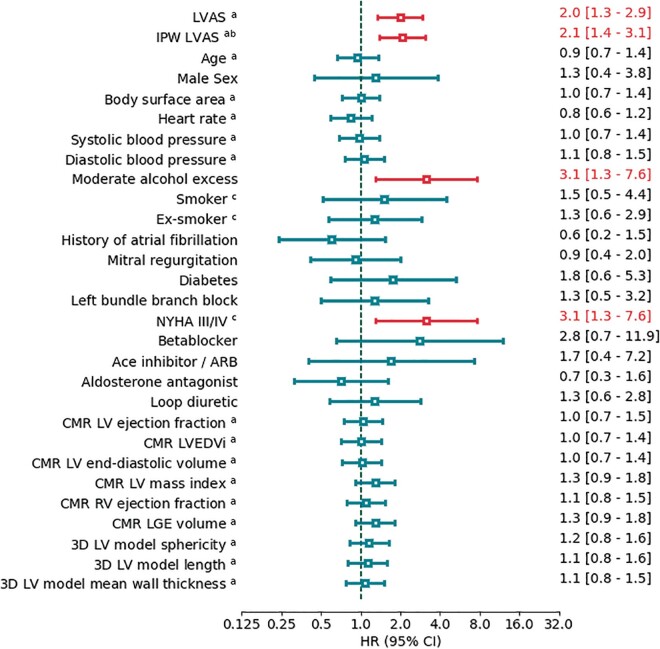
Association of baseline variables with earlier time to arrhythmic event in univariate cox regression. ^a^Per quartile. ^b^Propensity score includes age, sex, diabetes, moderate alcohol excess, NYHA III/IV, beta-blocker use, LV mass index, and LGE volume. ^c^Categorical variables, referent for smoker and ex-smoker is non-smoker, referent for NYHA III/IV is NYHA I/II. ARB, angiotensin receptor blocker; LGE, late gadolinium enhancement; LV, left ventricular; LVAS, left ventricular arrhythmic shape score; NYHA, New York Heart Association. Red = *P* < 0.05.

In a univariate time-to-event analysis of baseline variables (*Figure 6*), LVAS [hazard ratio (HR) = 2.0 per quartile, 95% confidence interval (CI) 1.3–2.9, *P* = 0.001], moderate alcohol excess (HR = 3.1, 95% CI 1.3–7.6, *P* = 0.012), and NYHA class III or IV (HR = 3.1, 95% CI 1.3–7.6, *P* < 0.012) were significantly associated with the arrhythmic endpoint. To test for potential confounding, a propensity score for LVAS was created using age and sex, as well as baseline variables with no missing data and *P* < 0.25 in univariate time-to event analysis (*Figure 6*). These variables were moderate alcohol excess, NYHA class III or IV, beta-blocker use and CMR LV mass index, and CMR LGE volume. Inverse probability weighting with the resulting propensity score reduced the Pearson correlations between LVAS and the selected baseline variables to 0 (Supplementary material online, *Figure S6*). Adjusting for the propensity score by inverse probability weighing, LVAS remained predictive of the arrhythmic endpoint (HR = 2.1 per quartile, 95% CI 1.4–3.1, *P* < 0.001). Sensitivity analysis with a covariate adjustment by the propensity score provided a similar result (Supplementary material online, *Figure S7*).

**Figure 7:**
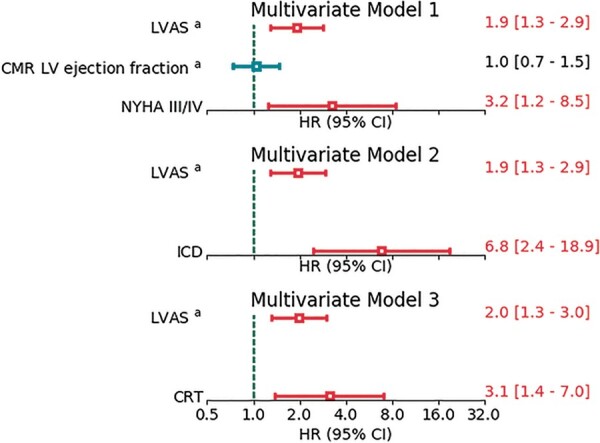
Association of LVAS with arrhythmic events after multivariate adjustment. ^a^Per quartile. LVAS, left ventricular arrhythmic shape score, Red = *P* < 0.05. ICD and CRT are time-varying covariates. CRT, cardiac resynchronization therapy; ICDs, implantable cardioverter-defibrillators.

In a multivariate time-to-event analysis (*Figure 6*), LVAS (HR = 1.9 per quartile, 95% CI 1.3–2.9, *P* = 0.01) and NYHA class III or IV (HR = 3.2, 95% CI 1.2–8.5, *P* = 0.016) were significantly associated with arrhythmic events in a model which included LV ejection fraction. Two further models were built to control for potential detection bias due to ICD implantation, and for potential confounding by CRT treatment (Models 2 and 3 in *Figure 7*). The association of LVAS with arrhythmic events was essentially unchanged in both models.

We further investigated the role of the 10 shape modes in a univariate analysis (Supplementary material online, *Figure S8*), and found that M6 (HR = 1.6 per quartile, 95% CI 1.1–2.3, *P* = 0.01) was significantly associated with the arrhythmic endpoint, with M5 being on the border to significance (HR = 0.7, per quartile, 95% CI 0.5–1.0, *P* = 0.05). Both M5 and M6 are accounted for in the LVAS.

## Discussion

In this study, we applied a novel computational anatomy approach for assessing the LV shape of DCM patients with LGE on CMR. We derived a novel shape score (LVAS) constructed from the geometrical modes of variation. Left ventricular arrhythmic shape was predictive of arrhythmic events in univariate time-to event analysis, and in multivariate analysis after adjustment for LV ejection fraction and NYHA class III/IV (Model 1), ICD receipt (Model 2), and CRT treatment (Model 3). We also confirmed the predictive value of LVAS after adjusting for a propensity score, further highlighting the independent nature of LVAS. This was in contrast to the two largest modes of shape variation, M1 and M2, which were highly correlated with LV functional and shape metrics, yet showed no significant prognostic value for arrhythmic events.

### Additional predictive power from a comprehensive three-dimensional morphological analysis

Our approach aimed to analyse the *complete* 3D LV morphology in a single analysis, thus exploiting the full breath of anatomical detail available in the CMR data. Previous approaches in ischaemic cardiomyopathy patients (ICM)^8^ and mixed cohorts^12^ have used predefined morphological and LGE-based measurements, such as sphericity index and wall curvature, to predict appropriate ICD therapy^12^ and sudden cardiac death.^8^ Our machine learning procedure automatically captured the most robust and relevant shape features, thereby eliminating the need for predefined geometrical metrics. Our findings confirm the improved discriminant power enabled by computational anatomy tools, which have been for example used previously to reveal the subtle signature of a premature birth.^13^

### Potential arrhythmogenic mechanisms underlying anatomical changes

Patients in the high-risk end of the LVAS distribution had an arrhythmic shape component characterized by a parabolic long-axis cross-section, as opposed to the ellipsoidal long-axis cross section at the low-risk end of the LVAS distribution (*Figure 2*). A plausible hypothesis is that the parabolic shape may be a sign of tissue remodelling and diffuse fibrosis processes that change the end-diastolic geometry, and at the same time make future ventricular arrhythmia more likely to occur. Previous research has shown an association between diffuse fibrosis and LV stiffness.^14^ A stiffer LV may lack a stretching response to an increase in LV pressure at end-diastole, potentially leading to a parabolic, high LVAS shape. This is in contrast to a compliant ventricle, which may more easily stretch into an ellipsoidal shape at end-diastole (i.e. low LVAS shape). At the same time, increased diffuse fibrosis may disturb electrical conduction in the LV,^15^ thereby contributing to re-entrant activations and ventricular arrhythmias. Further studies with parametric mapping could investigate this hypothesis. It is also possible that the arrhythmic shape is related to specific high-risk genetic DCM phenotypes,^16^ especially those involving FLNC genetic variants,^16^ which have noted basal-lateral wall dysfunction, in line with our observation of basal-lateral wall thinning in the arrhythmic shape mode (*Figure 4*).

In addition to LVAS being a potential marker of diffuse fibrosis, the parabolic LV shape it defines may also help to sustain re-entrant activity through increased tissue area at the base, providing a larger ‘effective electrical size’.^6^ As scar is most prevalent in basal regions in arrhythmogenic DCM,^17^ increasing the room that re-entrant wavefronts have to circulate in these key areas may provide an important mechanism of arrhythmogenesis in this population, defined and quantified by our LVAS metric.

### Risk stratification in dilated cardiomyopathy

Identifying which patients with DCM are at risk of future arrhythmia is a challenging problem, particularly relevant for the selection of patients for ICD therapy.^2^ Analysis of LGE-CMR images is a promising direction for risk stratification improvement, as the presence of LGE has been shown to herald an adverse prognosis.^3^ However, histological investigations have revealed the presence of up to 50% diffuse fibrosis in areas that are undetected by the common thresholding techniques applied to LGE-CMR images.^18^ Our study suggests that the LV shape, as quantified by LVAS, may be a useful arrhythmic risk predictor, potentially related to diffuse fibrosis. Previous studies have investigated phenotypic clustering,^19^ ECG QRS-width,^20^ LV entropy on LGE-CMR,^10^ and myocardial-LGE interface area on LGE-CMR.^11^ Given the diverse aetiology of non-ischaemic disease, it is likely that a variety of risk stratifying variables will be required to achieve accurate arrhythmia risk predictions in DCM patients. The potential utility of our shape-based arrhythmia predictor for DCM patients without LGE, or mixed ischaemic and non-ischaemic LGE, remains an open question.

### Study limitations

Our study is limited by its retrospective, observational design, and the single centre used in the study, and should be viewed as exploratory. Most importantly, our results lack validation in an external cohort and with prospective data. The limited sample size and number of events limited the number of potential confounders that could be adjusted for in multivariate analysis. We sought to mitigate these limitations with cross-validation methodology and propensity score adjustment. Implantable cardioverter-defibrillator programming was not guaranteed to be uniform, which presented a potential source of event detection bias. Lacking longitudinal data, we could not account for the effects of reverse remodelling due to CRT or other causes. Finally, we have also not assessed the potential role of electrophysiological tissue changes in our patients, and it is therefore possible that we have identified specific anatomical markers that are associated with electrophysiological remodelling (of ion channel dynamics and/or gap junctions) at more advanced stages of the disease.

## Conclusions

Left ventricular shape-based features, which describe the complex 3D anatomical patterns of remodelling in DCM with LGE, are promising independent risk predictors of ventricular arrhythmias and sudden cardiac death, which can potentially improve risk stratification models and procedures.

### Funding

This work was supported by a New Investigator Research Grant to M.J.B. from the Medical Research Council (MR/N011007/1), a Wellcome Trust Senior Research Fellowship (209450/Z/17/Z) to P.L., and the Norwegian Research Council via the ProCardio Center for Innovation [32481]. This research was further supported by the Welcome EPSRC Centre for Medical Engineering at King’s College London (WT 203148/Z/16/Z), the National Institute for Health Research (NIHR) Biomedical Research Centre (BRC) and CRF based at Guy’s and St Thomas’ NHS Foundation Trust and King’s College London, along with Imperial College London NIHR BRC, The Royal Brompton CRC and NIHR BRU, Alexander Jansons Foundation, and the BHF (FS/15/29/31492).

**Conflict of interest:** none declared.

### Data availability

Fully anonymized data will be shared where patient consent allows, on reasonable request to the corresponding author. Appropriate institutional data transfer agreements will be required. All anonymized LV geometrical meshes are available at: https://doi.org/10.6084/m9.figshare.11417295.

## Supplementary Material

euab306_Supplementary_Data
