# Supplementary material for: Left ventricular shape predicts arrhythmic risk in fibrotic dilated cardiomyopathy
Source: Europace. 2021 Dec 15;24(7):1137–47. doi: 10.1093/europace/euab306 (PMC9301973; doi:10.1093/europace/euab306)
Supplement: euab306_Supplementary_Data [file euab306_supplementary_data.zip › Supplementary_material.docx]

Supplementary Material

Left Ventricular Shape Predicts Arrhythmic Risk in Fibrotic Dilated Cardiomyopathy

Gabriel Balaban, Brian P. Halliday, Daniel Hammersley, Christopher A. Rinaldi, Sanjay K. Prasad, Martin J. Bishop*, Pablo Lamata*

*Joint senior authors

# Supplementary Methods

# LVAS Cross-validation and PCA mode Selection

The shape features that were most associated with the arrhythmic outcomes were found through a combination of two statistical techniques, Principle Component Analysis (PCA), and cross-validated Cox-Lasso regression. First, instead of using traditional descriptors of shape, that are heavily correlated and bulk (e.g. volume, length, thickness) we found a comprehensive description of 3D shape that gave us orthogonal and uncorrelated descriptors. We did this with PCA, and as a result we achieved a compact representation of shape in a very small number of variables. Instead of working with thousands of descriptors (e.g. the long list of nodes in the computational meshes) we worked with a reduced set of PCA modes, to reduce the risk of overfitting our prediction model. The cost of using a reduced set of PCA modes (1-10), was the potential loss of predictive anatomical information contained within the small PCA modes (11-156), which we did not consider in the Cox-Lasso model. The benefit of this choice was increased robustness, as PCA modes 1-10 accounted for the majority of the shape variance (Fig S3B).

Second, we choose a supervised machine learning technique that is very simple and robust, a Cox-Lasso regression that finds a linear combination of PCA modes. An important benefit of this choice is that the resulting linear combination is another mode of anatomical variation that can be represented and interpreted (see Fig.4 in main manuscript). The evaluation of the generality of our findings is thus based on cross-validation of the supervised machine learning task, i.e. the result of the optimal linear combination of the PCA modes. Candidate models were generated using a penalized Cox-lasso technique with all of the patient shape mode and outcome data. The L1 penalty parameter was varied so that a nested set of models was chosen, with each model containing one more shape variable than the previous model. For each model, we calculated the cross validated log-likelihood, using a leave-one out technique (1)⁠. The model with the maximal cross validated log-likelihood was chosen (Fig S4), which contained the shape modes M5, M6 and M10.

# Univariate Cox Regression with Missing Data

Several variables that were tested for an association with the arrhythmic end-point contained missing entries. In particular, smoking information (Smoker, Ex-smoker, Non-smoker) was missing for 8 cases (5.1%), History of atrial fibrillation for 23 cases (14.7%) and Mitral regurgitation for 3 cases (2%). For these variables univariate hazard ratios were estimated using multiple imputation with chained equations (MICE), implemented in the Python package AutoImpute. The categorical smoking variable was imputed with predictive mean matching, whereas the binary History of atrial fibrillation and Mitral regurgitation variables were imputed with logistic regression models trained on all baseline variables in Table 1, the event indicator, and the Nelson-Aalon estimate of the cumulative hazard function (2)⁠. 15 imputed datasets, corresponding to the highest percentage of missing data, were created using 4 MICE iterations per dataset. The hazard ratios and confidence intervals from each of the imputed datasets were combined into a single hazard ratio and confidence interval using Rubin’s rules (3)⁠.

# Propensity Score Calculation

Inverse probability weights (IPW), based on a propensity score, were calculated for the LVAS variable using Age, Sex, and baseline variables with p < 0.25 in univariate Cox regression for the arrhythmic end-point and complete data. These additional baseline variables were Moderate alcohol excess, NYHA III/IV, Beta-blocker, LV mass index, and LGE volume. The history of atrial fibrillation variable had p < 0.25 but was not considered due to missing entries. All other baseline variables with p < 0.25 in univariate Cox regression had complete data and were included in the analysis.

A non-parametric method was used to calculate the IPW (4)⁠. In brief the method solves an optimization problem that generates a set of weights which maximally de-correlate the target variable with the potential confounders, while maximizing the empirical likelihood of observing the data. We implemented the IPW method in Python and used the L-BFGS-B solver from Scipy to solve the optimization problem. Figure S5 (right) shows the distribution of the resulting IPW for all patients, and for the Event and No-Event patients. The mode of each distribution is very close to 1, and all weights value between 0.5-1.9. In Figure S5 (left) we visualise the Pearson correlations between the LGE metrics and the selected baseline variables. The Pearson correlation between the baseline variables and the LVAS was minor (< 0.15), and all of the correlations were reduced to 0 after reweighing.

# LGE Metric Calculations and LVAS correlation

LGE pattern metrics were calculated as part of a previous study (5)⁠ and are included here for a comparison with the LVAS score. In particular, the correlations between LVAS and the measured LGE metrics are relatively small (< 0.2, Figure S6). The individual metrics and their calculation are described in (5)⁠ and its supplement. A script for calculating the metrics from LGE MRI images is publicly available at [www.github.com/GabrielBalabanResearch/lgemri_scar_metrics](http://www.github.com/GabrielBalabanResearch/lgemri_scar_metrics).

# Short Axis Image Inconsistency Correction

Short axis images were inconsistent in 11 patients, which required some correction to create a consistent 3D image stack for geometrical model building. These inconsistencies included differences in image resolution, image size, or large slice misalignments. Differences in image resolution were resolved by resampling to the resolution of the majority of the short axis slices, whereas image size differences were handled by removing pixels in the larger images until all short axis slices shared the same size. Large slice misalignments were resolved by manually selecting the misaligned slices, and aligning the centres of their LV blood pool with a 2nd degree polynomial fitted to the blood pool centres of the remaining slices.

# Supplementary Results

# Fig S1: Timing of ICD implantations and arrhythmic events relative to baseline


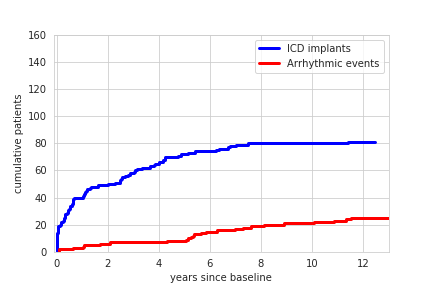


# Fig S2: CMR vs 3D model end-diastolic volumes

1.
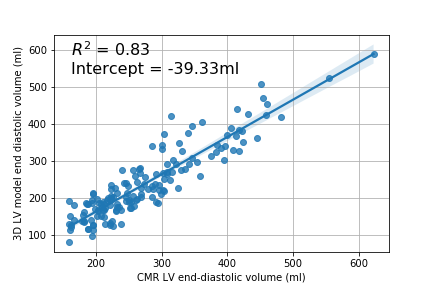

2. *The end-diastolic volumes from CMR and the 3-D models have a consistent linear relationship. The model intercept of -39 ml can be explained by 3D model volumes being lower due to valve plane image slices being excluded from the 3D models.*

# Fig S3: The first 10 modes of shape variation


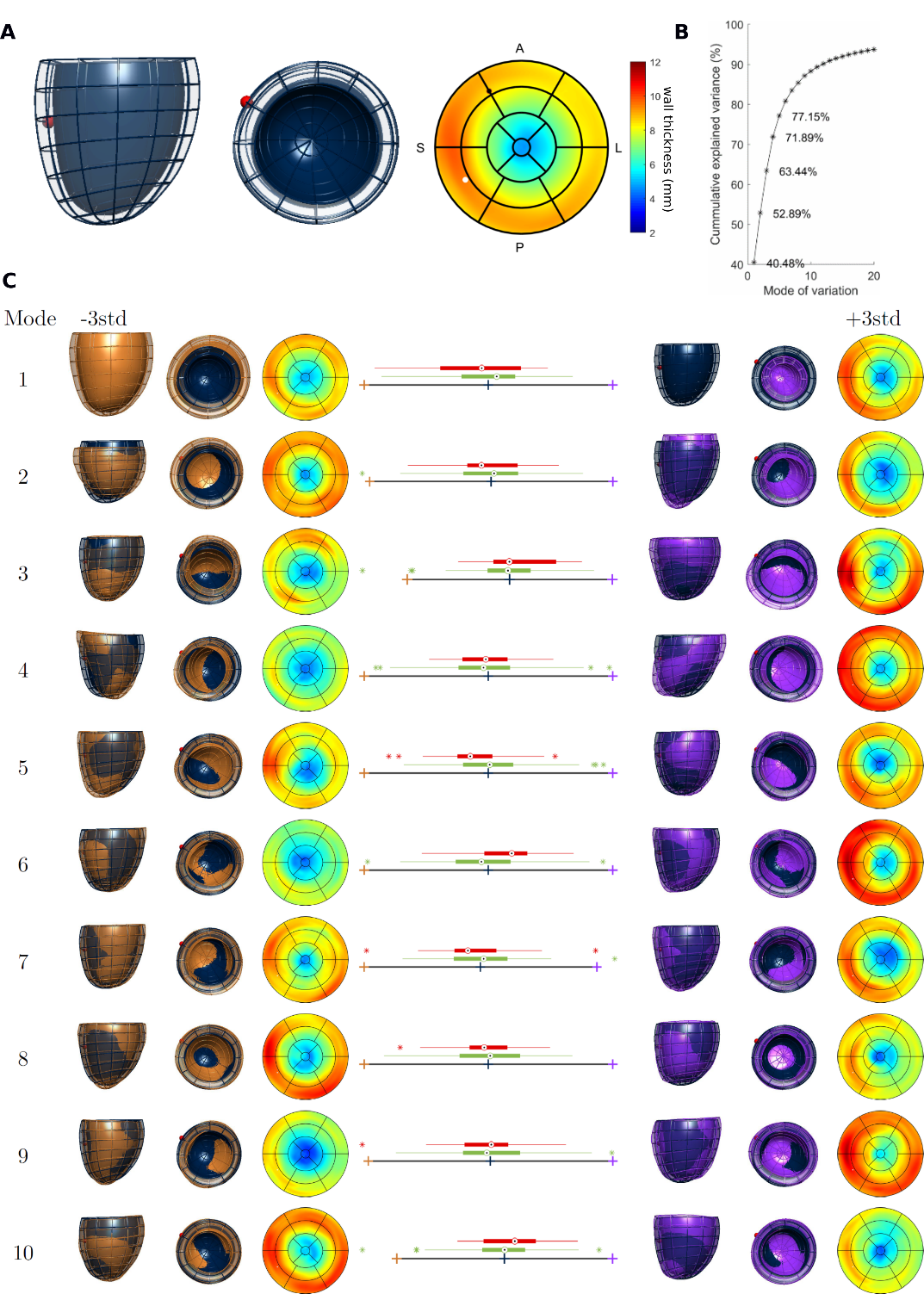


**Panel A**: Average anatomy (i.e. atlas) of the 156 cases with two views of the same 3D model in dark blue. The red sphere and white dot indicate the direction towards the RV landmark, in the 3-D geometries and wall thickness map respectively. **Panel B**: Cumulative variance explained by each mode of variation. **Panel C**: The first 10 independent modes of variation (M1 to M10). In all 10 cases, box-plots represent the no-event (green) and arrhythmic event (red) patient distributions in each mode, with the average at coordinate 0 represented by dark blue cross. In the 3D models, the average is the dark blue LV mesh overlaid on the orange and purple meshes. The orange (purple) cross and orange (purple) LV mesh represent −3SD (+3SD) for each mode of variation. Modes of variation represent changes in: 1= size (note +/- in axis is reversed with respect to Figure 2); 2= length linked to apex shift in the right to left direction; 3,4&5= apex shift in different combinations of the anterior to posterior or left to right directions, linked to wall thickness changes; Rest(6 to 10) = more subtle modes of anatomical change.

# Fig S4: Shape mode variable selection with penalized Cox-Lasso models

#
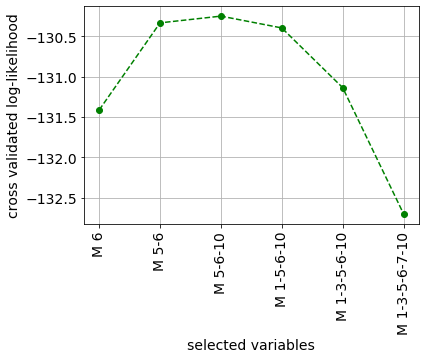


The variables of the models on the x-axis were selected using a penalized Cox-Lasso technique applied to all of the data. Going from left to right the regularization strength is decreased, and more complex models are selected. The model containing shape modes M5 M6 and M10 maximizes the cross validated log-likelihood.

# Fig S5: Spearman correlations between LVAS and LGE metrics


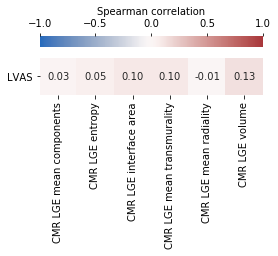


1. *All of the correlations between the LVAS score and the LGE metrics are <= 0.13. None of the correlations are statistically significant in a two- tailed t-test.*

#
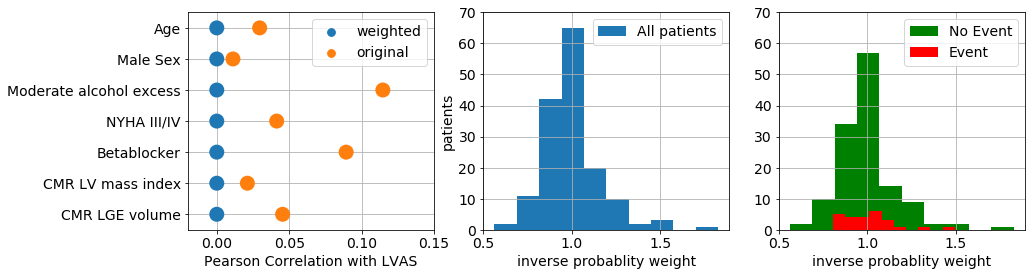
Fig S6: Inverse Probability Weight Distributions and Correlations

# *A) Pearson correlation values of LVAS with Age, Sex and variables associated with arrhythmic events at p < 0.25 in univariate cox regression and with no missing data. The correlations are reduced to 0 after the inverse probability weights are applied. B) Distribution of the inverse probability weights throughout the entire cohort, and in the Event and No-Event groups*

# Fig S7: Sensitivity analysis of propensity score adjustment, using the inverse probability weighing and covariate adjustment methods. *† Hazard ratios are per quartile.*


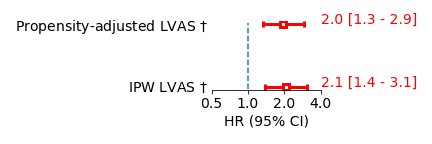


# Fig S8: Univariate Cox regression results for the first 10 PCA shape modes


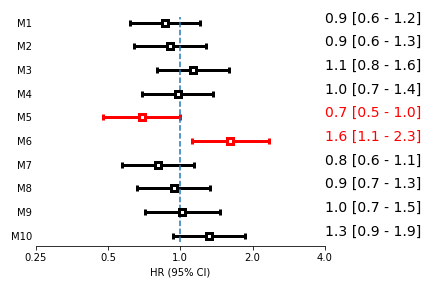


*Hazard ratios are per quartile. The hazard ratio of shapes mode M6 is statistically significant (P < 0.05), with M6 being on the border to significance (P = 0.05). Note that the shape mode axes can be reversed by changing the sign of the shape mode coefficients. M5, M6 can therefore be interpreted either as protective or hazardous associations.*

# Table S1: Patient history prior to baseline related to DCM Aetiology

|  | Cohort  N = 156 | Missing All | No event  N = 131 | Missing No Event | Event  N = 25 | Missing Event | p | Corr  LVAS |
| --- | --- | --- | --- | --- | --- | --- | --- | --- |
| History of myocarditis | 8 (7.4%) | 48 (30.8%) | 7 (7.4%) | 36 (27.5%) | 1 (7.7%) | 12 (48.0%) | 1 | 0.13 |
| History of alcohol excess | 28 (19.3%) | 11 (7.1%) | 24 (20.0%) | 11 (8.4%) | 4 (16.0%) | 0 (0.0%) | 0.79 | 0.02 |
| History of chemotherapy | 4 (3.4%) | 38 (24.4%) | 4 (3.9%) | 29 (22.1%) | 0 (0.0%) | 9 (36.0%) | 1 | -0.01 |
| Family history of DCM | 20 (14.4%) | 17 (10.9%) | 16 (13.7%) | 14 (10.7%) | 4 (18.2%) | 3 (12.0%) | 0.52 | -0.04 |
| History of inherited muscular disease | 1 (0.8%) | 36 (23.1%) | 1 (1.0%) | 28 (21.4%) | 0 (0.0%) | 8 (32.0%) | 1 | -0.07 |
| Peripartum DCM | 2 (1.4%) | 12 (7.7%) | 2 (1.7%) | 12 (9.2%) | 0 (0.0%) | 0 (0.0%) | 1 | -0.03 |

*Data are frequency (%). p-values are calculated with Fischer Exact tests using the available data. The final column shows the point-biserial correlation with the LVAS score using the available data. None of these correlations is statistically significant in a two-tailed t-test.*

# Supplementary References

1. Verweij PJM, Houwelingen HCVAN. Penalized Likelihood in Cox Regression. 1994;13:2427–36.

2. White IR, Royston P. Imputing missing covariate values for the Cox model. 2008;(April):4267–78.

3. Austin PC, White IR, Lee DS, van Buuren S. Missing Data in Clinical Research: A Tutorial on Multiple Imputation. Can J Cardiol. 2020;(May):1–10.

4. Fong C, Hazlettand C, Imai K. Covariate balancing propensity score for a continuous treatment: Application to the efficacy of political advertisements. Ann Appl Stat. 2018;12(1):156–77.

5. Balaban G, Halliday BP, Porter B, Bai W, Nygåard S, Owen R, et al. Late-Gadolinium Enhancement Interface Area and Electrophysiological Simulations Predict Arrhythmic Events in Patients With Nonischemic Dilated Cardiomyopathy. JACC Clin Electrophysiol. 2021;7(2):238–49.
